# Association study of candidate DNA-repair gene variants and acute graft versus host disease in pediatric patients receiving allogeneic hematopoietic stem-cell transplantation

**DOI:** 10.1038/s41397-021-00251-7

**Published:** 2021-10-28

**Authors:** C. R. S. Uppugunduri, P. Huezo-Diaz Curtis, T. Nava, M. A. Rezgui, V. Mlakar, S. Jurkovic Mlakar, N. Waespe, Y. Théoret, F. Gumy-Pause, F. Bernard, Y. Chalandon, J. J. Boelens, R. G. M. Bredius, J. H. Dalle, C. Nath, S. Corbacioglu, C. Peters, P. Bader, P. Shaw, H. Bittencourt, M. Krajinovic, M. Ansari

**Affiliations:** 1 CANSEARCH Research Platform in Pediatric Oncology and Hematology, Department of Pediatrics, Gynecology and Obstetrics, University of Geneva, Geneva, Switzerland; 2 Division of Pediatric Oncology and Hematology, Department of Pediatrics, Gynecology and Obstetrics, Geneva University Hospitals and University of Geneva, Geneva, Switzerland; 3 Charles-Bruneau Cancer Center, Sainte-Justine University Health Center (SJUHC), Montreal, QC Canada; 4 Childhood Cancer Research Group, Institute of Social and Preventive Medicine, University of Bern, Bern, Switzerland; 5 Clinical Pharmacology Unit, Sainte-Justine University Health Center (SJUHC), Montreal, QC Canada; 6 Division of Haematology, Department of Oncology, Geneva University Hospitals and University of Geneva, Geneva, Switzerland; 7 Paediatric Blood and Marrow Transplantation Program, University Medical Center Utrecht, Utrecht, The Netherlands; 8 Department of Paediatrics, Leiden University Medical Center, Leiden, The Netherlands; 9 Department of Hemato-immunology, Robert-Debre Hospital, Paris-Diderot University, Paris, France; 10 Department of Biochemistry, The Children’s Hospital at Westmead, Westmead, NSW Australia; 11 Department of Paediatric Haematology, Oncology and Stem Cell Transplantation, University Hospital of Regensburg, Regensburg, Germany; 12 Department of Pediatrics, St. Anna Children’s Hospital, Medical University of Vienna, Vienna, Austria; 13 Division for Stem Cell Transplantation and Immunology, Department for Children and Adolescents, University Hospital, Goethe University Frankfurt, Frankfurt, Germany; 14 The Cancer Centre for Children, The Children’s Hospital at Westmead, Westmead, NSW, Australia; 15 Department of Paediatrics, Sainte-Justine University Health Center (SJUHC), Montreal, QC Canada

## Abstract

Acute Graft versus Host Disease (aGvHD) grades 2–4 occurs in 15–60% of pediatric patients undergoing allogeneic haematopoietic stem-cell transplantation (allo-HSCT). The collateral damage to normal tissue by conditioning regimens administered prior to allo-HSCT serve as an initial trigger for aGvHD. DNA-repair mechanisms may play an important role in mitigating this initial damage, and so the variants in corresponding DNA-repair protein-coding genes via affecting their quantity and/or function. We explored 51 variants within 17 DNA-repair genes for their association with aGvHD grades 2–4 in 60 pediatric patients. The cumulative incidence of aGvHD 2–4 was 12% (*n* = 7) in the exploratory cohort. *MGMT* rs10764881 (G>A) and *EXO* rs9350 (c.2270C>T) variants were associated with aGvHD 2–4 [Odds ratios = 14.8 (0 events out of 40 in rs10764881 GG group) and 11.5 (95% CI: 2.3–191.8), respectively, multiple testing corrected *p* ≤ 0.001]. Upon evaluation in an extended cohort (*n* = 182) with an incidence of aGvHD 2–4 of 22% (*n* = 40), only *MGMT* rs10764881 (G>A) remained significant (adjusted HR = 2.05 [95% CI: 1.06–3.94]; *p* = 0.03) in the presence of other clinical risk factors. Higher *MGMT* expression was seen in GG carriers for rs10764881 and was associated with higher IC50 of Busulfan in lymphoblastoid cells. *MGMT* rs10764881 carrier status could predict aGvHD occurrence in pediatric patients undergoing allo-HSCT.

## Introduction

The most frequent immunological complication after allogeneic haematopoietic stem-cell transplantation (allo-HSCT) is acute Graft versus Host Disease (aGvHD), occurring in 15–60% of transplants in children [1]. In patients receiving HSCT from HLA-identical siblings, the long-term survival rates with aGvHD grades III–IV are below 30% [2]. aGvHD begins with host normal tissue damage by the conditioning regimens that causes pro- and anti-inflammatory cytokine secretion which subsequently activate the host antigen presenting cells (this being phase 1 of the pathobiology of aGvHD) [3]. Thus, the intensity or type of conditioning regimen is determined as one of the donor-independent risk factors for aGvHD [4, 5].

Busulfan (BU) is frequently used for conditioning children prior to allo-HSCT [6]. BU is a bifunctional alkylating agent (AG) commonly administered with other alkylating agents like cyclophosphamide (CY) or the purine analog Fludarabine (FLU) [7]. BU and other AGs mediate their cytotoxicity by damaging the DNA through formation of covalent linkages between the alkyl groups, mainly the N^7^ position of guanine, while the N^3^ position of cytidine and O^6^ of guanine also serve as nucleophiles [8]. These covalent modifications lead to inter- or intra-strand DNA crosslink formation, which affects the genomic integrity and causes deleterious consequences during DNA replication. That effect is observed in tumor cells but also in normal cells, the latter being linked to the treatment-related toxicities (TRTs) such as aGvHD. Hence, variants related to BU metabolism such as GSTA1*B [9] and *GSTM1-null* [10] were described as risk factors for aGvHD. Other genetic polymorphisms within immunological pathways were also described as risk factors [11], some of them in a pediatric population [12].

The DNA damage caused by AG is repaired by various DNA-repair pathways [8, 13] including base excision repair (BER), mismatch repair (MMR) and homologous recombination or by nonhomologous end joining. Other mechanisms include demethylation of guanine residues by O6-methylguanine-DNA methyltransferase (MGMT) and MMR of small insertions and modifications by identifying the damaged base with the help of Mut L homologue-1 protein [14]. Genomic predictors of interindividual differences in response to DNA damaging agents have previously been demonstrated [15]. Studies have implicated the role of genetic variants and altered expression of genes in the DNA-repair pathways particularly BER and MMR in determining treatment outcomes of AGs [16]. However, less is known about their role in determining clinical outcomes of BU-based conditioning in a pediatric allo-HSCT.

We hypothesized that children receiving allo-HSCT with efficient DNA-repair ability are at reduced risk of developing aGvHD by diminishing the activation of Phase 1 of the pathophysiology of aGvHD. As the DNA damage caused by cross-linking agents like BU is complex and may involve one or more of the above-mentioned pathways; in this study candidate genes from key pathways were therefore investigated as possible biomarkers for aGvHD. Selected variants (list of the candidates selected and criteria for the selection of variants are provided in the methods section) among the genes coding for key proteins of demethylating repair pathways, BER pathway genes or double-strand break repair pathways were selected for the investigation [8, 13, 14, 16].

## Results

The characteristics of the study subjects in the exploratory cohort (*n* = 60) and extended cohort (*n* = 187) are given in Table 1. The incidence of aGvHD 2–4 was 12% (*n* = 7) in the exploratory cohort and 22% (*n* = 40) in the extended cohort.

**Table 1:** Characteristics of the study subjects.

**Characteristics**	**Exploratory** **(*N*** **=** 60)		**Extended** **(*N*** = 182)		***p*** **value for comparison between cohorts**	**Covariates included in multivariate analysis in extended analysis**
	***N***	**%**	***N***	**%**		
Gender					0.46	
Male	28	47	96	53		Not included
Female	32	53	86	47		
GSTA1 phenotype					0.32	
Rapid and normal metabolizers	53	88	149	82		Rapid and normal metabolizers
Slow metabolizers	7	12	33	18		Slow metabolizers
Diagnosis					1.00**	
Acute lymphoid leukemia	2	3	22	12		
Acute myeloid leukemia	18	30	49	27		
Myelodysplastic syndrome	16	27	32	18		
Myeloproliferative syndrome	1	2	7	4		
*Total malignancies*	37	62	110	60		Total malignancies
Bone marrow failure	0	0	2	1		
Hemoglobinopathies	8	13	22	12		
Immunodeficiency	8	13	32	18		
Metabolic disease	3	5	9	5		
Hemophagocytic syndrome	4	6	7	4		
*Total non-malignancies*	23	38	72	40		Total non-malignancies
HLA compatibility					0.77	
Mismatch-unrelated donor	22	37	64	35		MMUD
Mismatch-related donor	2	3	8	4		MMRD
Matched unrelated donor	13	22	51	28		MUD
Matched related donor	23	38	59	32		MRD
Stem cell source					0.06	
Bone marrow	26	42	74	41		Bone marrow
Cord blood	33	56	81	45		Cord blood
Peripheral blood	1	2	27	15		Peripheral blood
Myeloablative conditioning					<0.05***	
BU/CY/MEL	0	0	12	7		
BU/CY/VP16*	5	11	9	5		
Total number of three alkylating agents	5	11	21	12		Three alkylating agents
BU/FLU/Thio	0	0	7	4		
BU/CY	55	89	95	52		
BU/FLU/CY	0	0	3	2		
BU/MEL	0	0	1	1		
BU/FLU/MEL	0	0	21	12		
Total number of two alkylating agents	55	89	127	68		Two alkylating agents
BU/FLU or FLU/BU	0	0	34	20		One alkylating agent
Serotherapy						
No	15	23	65	36	0.038	No
ATG	47	71	114	63		Yes
AL	0	0	3	2		
GvHD prophylaxis						
Missing data	0	0	1	1	NC	
Steroids alone	0	0	2	1		Not included
Cyclosporine + steroids	33	56	48	26		
Cyclosporine + MTX	27	44	72	40		
CSA + MMF	0	0	12	7		
Cyclosporine alone	0	0	39	21		
Total number of CSA-based prophylaxis			171	94		Total CSA-based prophylaxis
Tacrolimus	0	0	3	2		
Tacrolimus + MTX	0	0	4	2		
Tacrolimus + MMF	0	0	1	1		
Total number of TAC-based prophylaxis			8	4		Total TAC-based prophylaxis
	Median	Range	Median	Range		
Age (years)	6.4 (6.3)	0.1–19.9	5.6 (5.8)	0.0–23.7	>0.05	Not included
cumAUC (mg.H/L)	63.1 (7.9)	40.82–84.82	63.8 (13.3)	28.8–110.52	>0.05	cumAUC (mg.H/L)
BU Day 1 AUC (mg.H/L)^a^	12.9 (3.9)	7.3–28.8	13.10 (4.3)	5.90–29.30	>0.05	BU day 1 AUC (mg.H/L)

*GSTA1 genotyping was either performed according to the previously described procedures [9] or with sanger sequencing of the promoter region. GSTA1 metabolic status was based on reporter-gene assays and PK data as described in Ansari et al. [9]. *BU/CY/VP16 was included in this group due to its reported higher toxicity equal to three alkylating agents.

*BU* Busulfan, *CY* Cyclophosphamide, *MEL* Melphalan, *VP16* etoposide, *ATG* anti-thymocyte globulin, *AL* alemtuzumab, *MTX* methotrexate, *MMUD* mismatch-unrelated donor, *MMRD* mismatch-related donor, *MUD* matched unrelated donor, *matched related donor* matched related donor, *NC* *p* value not calculated as the distribution of several heterogenous prophylactic combinations exists with no patients receiving this combination in one of the cohorts.

**p value for the distribution of malignancies versus non-malignancies.

***p value for the distribution of single versus two versus three alkylating agents’ usage.

^a^BU 1^st^ Day AUC s were presented irrespective of the dosing schedule used in the patients (either four times daily for all the four doses combined or once daily for one dose). AUCs are presented to reflect the exposure of BU in each patient which is a derived pharmacokinetic parameter from observed clearance and administered doses.

### DNA-repair genetic variants and aGvHD

**Fig. 1:**
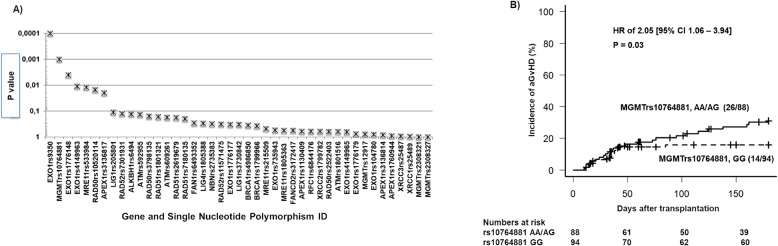
Association of DNA repair candidate genetic variants with aGvHD (grades2-4) in pediatric allo-HSCT. **A** Clinical association analysis between DNA-repair SNP variants and aGvHD 2–4 in the exploratory cohort: forty-three SNPs were carried forward for statistical analysis. Association analyses between genotype (additive or dominant models) against aGvHD 2–4 were tested using an *X*^2^ test (Fishers exact test, two sided). Acute GVHD incidences in this cohort were 12%. In the *x*-axis, the gene and SNP identifications are given and in the *y*-axis their significance for association with aGvHD 2–4 are shown as *p* values. **B** Clinical association analysis between *MGMT* rs10764881 and aGvHD 2–4 in the extended cohort. Cumulative incidence of acute Graft versus Host Disease (aGvHD 2–4) in the extended sample (*N* = 182) using competing risk analysis and Cox-regression analysis to calculate the Hazard ratio (HR). Results plotted for *MGMT* rs10764881 genotype group AA and AG versus GG. The number of patients with aGvHD 2–4 /total number of patients in each group is provided on the plot along with p value and HR for this analysis. The numbers at risk for developing aGvHD 2–4 at each time interval on the *x*-axis is mentioned below the plot.

Hardy–Weinberg equilibrium (HWE) *p* values and minor allele frequency (MAF) data for each SNP is presented in Table 2. Five SNPs were found to be non-polymorphic in our sample set (*ALKBH1* rs17825440; *BRCA1* rs28897687; *FANCD2* rs9845756; *NBN* rs1805794; *RAD52* rs7310449). Another three SNPs did not pass at the genotyping stage due to unreliable amplification of product (*APEX1* rs4585; *BRCA1* rs28897687; *NBN* rs1805800). Forty-three SNPs were carried forward for statistical analyses. Association analyses between genotype (additive or dominant model) against aGvHD 2–4 are illustrated in Fig. 1A. *EXO* rs9350 and *MGMT* rs10764881 showed significance (multiple testing *p* value ≤0.001, with an odds ratio of 11.5 (95% CI: 2.3–191.8) and 14.8 (0 events out of 40). From the extended analysis of these SNPs with aGvHD, only *MGMT* rs10764881 (*p* = 0.03) remained significant (Fig. 1B).

**Table 2:** Description of the selected 51 genetic variants from 17 candidate DNA-repair genes.

Gene symbol, name, and *DNA-repair pathway*		Chromosomal location	dbSNP ID	Nucleotide Change	Amino Acid change, or UTR	Functionality Prediction	HapMap (CEU)^a^ MAF (%)	1000 Genomes (EUR)^b^ MAF (%)	MAF in this study (%)	HWE *p* value
*ALKBH1*	Alkylation Repair Homolog 1, ***De-Alkylation pathway***	14q24.3	rs17825440	T>C	Non-syn, M135I	Tolerated	4	2	0	–
			rs6494	A>T	Non-syn M324L	Tolerated	26	24	16	0.74
*APEX1*	*(APEX nuclease (multifunctional DNA-repair enzyme) 1)* ***Base Excision Repair Pathway***		rs1760944	T>G	Utr-5^1^	No direct binding	43	35	46	0.66
			rs3136814	C>A	Utr-5^1^	Tolerated	3	4	4	0.002
			rs3136817	C>T	Intron	No miRNA binding	22	29	19	0.23
			rs1130409	G>T	Non-syn, D148E		46	49	44	0.01
			rs4585	G>T	utr-3^1^		47	38	–	–
*ATM*	Ataxia Telangiectasia Mutated, ***Homologous recombination pathway***	11q22–q23	rs592955	A>C	utr-5^1^	No direct binding	43	38	49	0.8
			rs609261	T>C	utr-5^1^	Possibly damaging	49	38	49	0.9
			rs1801516	T>C	non-syn, D1853N	Tolerated	17	16	8	0.3
*BRCA1*	Breast Cancer 1, Early Onset, ***Homologous recombination pathway***	17q21.31	rs1799966	C>T	Non-syn, S1613C	Damaging	34	35	32	0.38
			rs28897687	C>A	Non-syn, N1236K	Damaging	1.1	0	–	–
			rs4986852	T>C	Non-syn, S1040N	Damaging	5	2	0	–
			rs4986850	T>C	Non-Syn, D693N	Damaging	10	8	4	0.05
*EXO1*	Exonuclease 1, ***Homologous recombination and Mismatch repair pathways***	1q42–q43	rs1776177	C>T	Non-syn,	Not found	49	46	48	0.74
			rs1776179	C>T	Non-syn	Not Found	27	31	27	0.15
			rs735943	A>G	Non-syn, H354R	Tolerated	42	42	34	0.302
			rs4149963	T>C	Non-Syn, T439M	Tolerated	7	8	8	0.004
			rs4149965	A>G	Non-Syn, V458M	Tolerated	30	25	12	0.94
			rs1047840	A>G	Non-syn,	Not found	39	37	43	0.78
			rs1776148	A>G	Non-Syn, E670G	Tolerated	35	34	29	0.401
			rs9350	T>C	Non-Syn, P757L	Damaging	15	15	25	0.186
*FAN1*	Fanconi-Associated Nuclease 1 ***Homologous recombination pathway***	14q11.2	rs6493352	T>C	Non Syn, R648H	Tolerated	17	18	20	0.58
*FANCD2*	Fanconi Anemia, Complementation Group D2 ***Homologous recombination pathway***	3p25.3	rs9845756	T>C	Utr-5^1^	Possibly damaging	20	13	0	–
			rs3172417	T>C	Utr-3^1^		39	45	23	0.17
*Lig1*	Ligase I, DNA, ATP-Dependent, ***Base Excision Repair Pathway***	19q13.33	rs20580	A>G	Utr-5^1^	No direct binding	46	49	42	0.72
			rs3730842	C>T	Coding-syn	Not found	13	10	20	0.56
*Lig4*	ligase IV, DNA, ATP-dependent, ***Base Excision Repair Pathway***	13q33.3	rs1805388	A>G	Missense, T9I	Damaging	19	16	15	0.84
*MGMT*	O-6-Methylguanine-DNA Methyltransferase,***De-Alkylation pathway***	10q26.3	rs10764881	A>G	Near-gene-5^1^	No direct binding	37	30	19	0.61
			rs12917	T>C	Non-syn, L115F	Damaging	10	13	18	0.90
			rs2308321	G>A	Non syn, I174V	Tolerated	16	13	7	0.49
			rs2308327	G>A	Non-syn, K209R	Tolerated	9	13	10	0.34
			rs113813075	C>A	Utr-5^1^		5	6		
*MRE11*	Meiotic Recombination 11 Homolog A, ***Homologous recombination pathway***	11q21	rs215509	C>T	Utr-3^1^		32	12	33	0.67
			rs533984	A>G	Intron		39	40	48	0.03
			rs1805363	T>C	Utr-5^1^		9	8	8	0.40
*NBN*	Nijmegen Breakage Syndrome 1, ***Homologous recombination pathway***	8q21.3	rs1805800	T>C	Utr-5^1^	Not direct binding	28	30	–	–
			rs1805794	G>C	Non-syn, E185Q	Tolerated	31	30	0	–
			rs2735383	G>C	Utr-3^1^		32	29	37	0.06
*RAD50*	RAD50 homolog, ***Homologous recombination pathway***	5q31.1	rs3798135	T>C	Intron		21	19	25	0.30
			rs2522403	C>T	Intron		22	19	31	0.05
			rs10520114	G>A	Intron		22	18	16	0.27
*RAD51*	RAD51 Recombinase, ***Homologous recombination pathway***	15q15.1	rs2619679	T>A	Utr-3		47	49	42	0.23
			rs7180135	G>A	Utr-3	Not found	47	43	27	0.55
			rs1801321	T>G	Non-syn,		47	42	27	0.81
*RAD52*	RAD52 Homolog ***Homologous recombination pathway***	12p13.33	rs7310449	C>T	Utr-3^1^		44	42	0	–
			rs7301931	C>T	Utr-3^1^		49	43	45	0.40
			rs11571475	G>A	Utr-3^1^		13	13	11	0.29
*RFC1*	Replication factor C, ***Mismatch Repair pathway***	4p14-p13	rs6844176	C>T	Intron		38	43	48	0.38
*XRCC1*	X-Ray Repair Complementing Defective Repair In Chinese Hamster Cells, ***Base Excision Repair Pathway***	19q13.2	rs25489	T>C	Missense, R280H	damaging	10	5	3	0.23
			rs1799782	A>G	Missense, R194W	damaging	12	5	12	0.43
			rs25487	T>C	Missense, Q399R	damaging	23	36	26	0.78

^a^CEU-Utah residents of Northern and Western European Ancestry.

^b^EUR-European population including both Finnish and non-Finnish European subpopulations.

The in silico functional evaluation for the non-synonymous genetic variants were predicted using four different tools SIFT (https://sift.bii.a-star.edu.sg), Polyphen (http://genetics.bwh.harvard.edu/pph2/), SNPs3D (http://www.snps3d.org), and PANTHER (http://www.pantherdb.org). The functional importance of the SNPs within 5′ flanking regions was predicted by looking at potential transcriptional binding sites, which may affect transcription, using the MatInspector tool (www.genomatx.de). The same approach was performed for SNPs within 3′ UTR looking for miRNA sites using TargetScan Human 5.1 (http://www.targetscan).

Multivariable analysis (Table 3), adjusting for known risk factors, indicated that *MGMT* rs10764881 allele A is an independent risk factor for aGvHD 2–4 (2.05 [95% CI: 1.06–3.94]; *p* = 0.03). Altogether with no serotherapy administration (HR 2.11 [95% CI: 1.08–4.14]; *p* = 0.03), higher 1st day BU AUC (HR 1.08 [95% CI: 1.01–1.15]; *p* = 0.03) and HLA mismatch (HR 1.97 [95% CI: 0.90–4.3; *p* = 0.08) remained within the model as risk factors. Multinomial regression examining *MGMT* rs10764881 with aGvHD severity demonstrated that the risk tended to be higher with severe grades of aGvHD, when patients carried the AA or AG genotypes. However statistical significance was not reached for determining an increased risk between aGvHD 1 vs. aGvHD 2–4 based on the genotype (*p* = 0.3, see Supplementary Table 1). *MGMT* rs10764881 was not associated with relapse post-transplant in patients with malignancies (data not shown).

**Table 3:** Multivariable Cox Regression of aGvHD 2–4 (*n* = 182).

Covariates	HR	95% CI		*P* value (multivariable)
		Lower	Upper	
*MGMT*				0.03
rs10764881 GG	1			
rs10764881 AA and AG	2.05	1.06	3.94	
*HLA compatibility*				0.09
MRD	1			
MUD, MMUD, MMRD	1.97	0.90	4.28	
Serotherapy				0.03
ATG	1			
No serotherapy	1.08	1.01	1.15	
Day 1 BU AUC	2.11	1.08	4.14	0.03

Variables included in the analysis (backward stepwise conditional cox-regression analysis, removing variables with *p* > 0.2); GSTA1 (rapid and normal metabolizer groups vs. slow metabolizer group); *MGMT* rs10764881 (GG vs. AA/AG); diagnosis (malignant vs. non-malignant); HLA matching (MRD vs. MUD, MMRD, MMUD); stem-cell source (bone marrow vs. peripheral blood stem cells vs. cord blood); chemotherapy (one alkylating vs. two alkylating agents or three or with VP16); serotherapy (ATG vs. no serotherapy); Day 1 BU AUC (mg × H/L)as a continuous variable harmonized for the dosing schedule (1 × daily or 4 × daily).

### *MGMT* mRNA expression pre- and post-busulfan exposure

*MGMT* mRNA expression demonstrated no significant change from basal levels (data not shown). Nevertheless, *MGMT* rs10764881 showed a change in expression levels irrespective of the BU treatment (*p* = 0.01 pretreatment and 0.03 posttreatment; Supplementary Fig. 1).

#### Cell viability studies on HAP1 *MGMT* knockout cell lines and lymphoblastoid cells (LCLs)

There was no significant difference in inhibitory concentration 50 (IC50) value between HAP1 *MGMT* knockout cells (mean IC50 = 102.21 µM ± 6.4 µM) and HAP1 parental cells after BU exposure (mean IC50 = 117.68 µM ± 16.7 µM) (Supplementary Fig. 2). However, we observed significant differences (*p* = 0.02) in the BU IC50 values between LCLs carrying “GG” and “AG, AA “genotypes for rs10764881 (Supplementary Fig. 3).

#### Dual luciferase reporter-gene assays

**Fig. 2:**
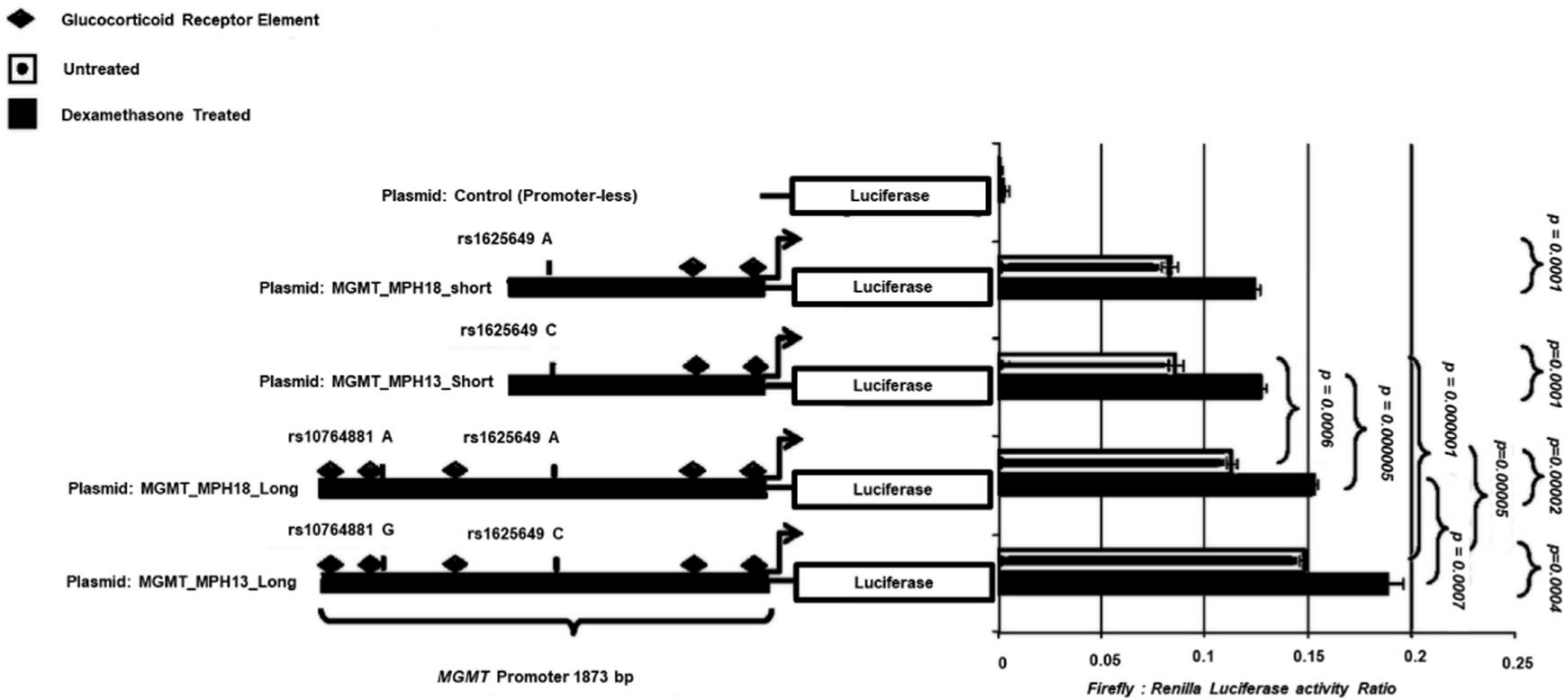
Reporter-gene assay on primary keratinocyte cells treated with and without dexamethasone. Site-specific mutations for *MGMT* were designed dependent on the SNP of interest in the promoter region. Two fragments of 785 bp (short constructs), both excluding variant rs10764881, but including another SNP, known as rs1625649 which is in high LD with rs10764881 (*D*′ = 0.99 and R2 = 0.84) were designed. The two other fragments are longer, 1873bp, one that includes rs10764881 allele G and the other with rs10764881 allele A. All four fragments were cloned into pGl4.10 in front of the firefly luciferase gene. Human epidermal primary keratinocytes (HEK cells) were co-transfected with each of the pGL4.10 *MGMT* constructs and the pRL-SV40 vector that codes for Renilla luciferase for transfection control and normalization. Promoter-less pGL4.10 plasmid was used to determine baseline expression. Measurement of Luciferase and Renilla activity was determined by Dual luciferase assays. With the use of the MatInspector tool, a Glucocorticoid responsive element (GRE) was located near to variant rs10764881 and other areas within the plasmid construct region. Thus, to understand whether the enhancing effects are related to corticosteroids, HEK cells transfected with the gene reporter plasmids were stimulated with 0.1 µM dexamethasone (Sigma, D8893) for 15 h and Luciferase expression examined as previously and compared to the non-treated. Difference in promoter activity between the plasmid constructs was assessed by t-test.

There was no significant differences observed in expression levels between the short plasmid construct containing alleles A and C of variant rs1625649 that is in strong LD (*r*^2^ = 0.85–1.0 in Europeans), with rs10764881, (*p* = 0.53) (Fig. 2). However, the longer plasmid construct containing variant rs10764881 allele G differed significantly from the plasmid construct containing variant rs10764881 allele A (*p* = 0.000005) as well as from the shorter fragment without rs10764881 (*p* = 0.000001) suggesting the presence of an enhancer element near to this SNP, which shows increased dependency with the presence of allele G.

### Dexamethasone-mediated activation of h*MGMT* promoter

Exposing the cells to dexamethasone increased protein expression of luciferase in all the plasmids compared to their non-treated plasmid construct (*p* < 0.0005). The highest response was seen from the plasmid construct containing variant rs10764881 allele G compared to the other treated constructs (*p* < 0.0007) (Fig. 2).

### Electrophoretic mobility shift assays

Electrophoretic mobility shift assays (EMSA) results showed that a shift is established when nuclear protein is added at varying concentrations to the predicted GRE probe (Supplementary Figs. 4, 5). The intensity of the shift diminished when unlabeled competitive probes were added at 100 times higher concentration, indicating that the DNA-protein binding occurs with the predicted probe region. Furthermore, interaction appears stronger when allele G is present in the predicted probe.

## Discussion

We showed an association of a variant in *MGMT* (rs10764881, G>A) with aGvHD 2–4 incidence, even after adjusting for other known risk factors (serotherapy, BU AUC and HLA incompatibility). A trend in association of AA and AG genotype carriers at rs10764881 with severity of aGvHD was also seen (no aGvHD, 35% vs. aGvHD grade 1, 55% vs. aGvHD 2–4, 66%). These observations suggest efficient DNA damage repair due to increased MGMT expression and activity in GG carriers. Minor allele (“A”) frequencies of rs10764881 among different ethnicities are similar (∼25–30%) except for the African population (<10%) indicating the utility of this genetic marker among non-African ethnicities (Supplementary Table 2).The only other clinical association study that has investigated DNA-repair genes in relation to aGvHD, assessed BER pathway genes and reported a significant association with a variant in *RFC1* (rs6844176) in a mixed cohort of adult and pediatric populations [16]. This variant was not significantly associated with aGvHD in our exploratory cohort. Thus, to the best of knowledge, this is the first report that has evaluated candidate DNA-repair gene variants and their association with aGvHD after allo-HSCT in children. This report unfolds the role of DNA-repair pathway gene candidate genes as a biomarker for stratification of patients at a higher risk of developing aGvHD post HSCT. Oligogenic risk score development may include DNA-repair gene variants along with other reported genetic risk factors in immunological mediators for e.g., interleukin 1 [11], interferon-gamma, interleukin 10, and TGF-β [17] or busulfan metabolic pathway [9] to evaluate performance for the prediction of aGvHD risk post HSCT.

Understanding the effect of DNA-repair gene variation on normal tissues could also be beneficial for assessing the risk of TRTs [13]. *MGMT* encodes the DNA-repair protein *O*^6^-alkylguanine DNA alkyl transferase. It has been studied extensively in association with methylating-agent resistance [18]. Earlier research has mainly focused on evaluating the inhibition of MGMT to augment therapy with alkylating agents. However, Phase I trials showed that inhibitors that inactivate MGMT, improved the efficacy of BCNU (1,3-bis(2-chloroethyl)-1-nitrosourea) on tumor cells but were associated with more systemic toxicity [19], indicating *MGMT* expression is also vital for protecting from DNA damage in normal cells. BU preferentially induces N^7^, *N*^3^ guanine, and *N*^3^ adenine lesions and MGMT is known to repair lesions of alkylation reactions at oxygen sites, such as O^6^ of guanine. No significant change in the IC50 values of BU in the absence and presence of MGMT in HAP1 cells, indicating its limited role in determining the cytotoxicity caused by BU. However, significant differences in IC50s of BU was observed between LCLs carrying different genotypes for rs10764881 in *MGMT*. GG carriers exhibited higher BU IC50 values compared to AA and AG carriers (Supplementary Fig. 3). However, this cannot entirely be attributed to rs10764881 genotypes, as several other gene variants at the same time were also associated with changes in BU IC50 in the LCLs. Multiple testing correction was not applied due to the limited number of the samples (*n* = 58). However, LCLs serve as a good model for investigating the association of potential pharmacogenetic markers. They represent unrelated individuals with the marker of interest. Thus, irrespective of the causative effect, *MGMT* rs10764881 genotypes could identify cells that are sensitive to BU or resistant to BU defined based on IC50 values. Interestingly, this SNP is not in linkage disequilibrium (LD) with any SNP in the SNPs in the MGMT exonic region, however it is in strong LD with rs1625649 (R2 > 0.85–1.0) in Europeans (except for Finnish population) and Americans, (specifically Mexican ancestry and Peruvian in Lima) and South East Asians (only in Punjabi in Lahore, Pakistan). A better progression free survival was seen in glioblastoma patients carrying rs1625649 “AA” genotypes which was in turn associated with lower *MGMT* expression [20] and was also shown to reduce the expression of *MGMT* as a part of investigated promoter haplotypes [21]. Thus, the observed decreased *MGMT*expression in rs10764881 AG, AA carriers can be partially explained by AA and AC genotypes at rs16265649 locus and as a result of the interaction between these two loci as demonstrated in the luciferase assays in this report (Fig. 2). The allele frequencies of rs1625649 are given in Supplementary Table 2. In addition to BU induced DNA damage, co-administering agents with BU such as CY and its metabolites also contribute to the DNA damage and thus play a role in the occurrence of aGvHD. MGMT was shown to be involved in repairing damage caused by acrolein, a cyclophosphamide (CY) metabolite [22]. Thus, the association observed in this study might be due to an indirect effect through the interaction of BU with combination chemotherapy. For e.g., depletion of glutathione (GSH) reserves by BU conjugation may reduce the elimination of CY’s metabolite acrolein, etoposide’s metabolite quinone or melphalan in turn increasing tissue damage [23], which MGMT could repair. Our results demonstrate that higher 1st day BU exposure increases the risk of aGvHD incidence. Though, direct proportional relationship between MGMT and GSH is well known [24], this needs to be further explored the within an HSCT setting in relation to the conditioning regimen. O6-methyl guanine adducts in the absence of MGMT activity could generate point mutations, mismatching base pairs and lead to the formation of DNA double-strand breaks (DSB) [25]. Thus, the inter-play between MGMT repair and other DNA-repair pathways in elucidating the cell death and toxicity of alkylating agents used in HSCT conditioning also need to be addressed in future.

Genome wide e-QTL analysis, showed difference in mRNA expression in relation to rs10764881 across diverse human tissues, where allele G demonstrated higher expression profiles [26]. These findings were depicted in LCLs mRNA expression experiments in this report. Our reporter-gene assays indicate that the region further upstream (−700 to −1873) might contain an enhancer element dependent on allele G and to a lesser extent on allele A. MatInspector tool predicted GREs in the human *MGMT* promoter (Supplementary Fig. 4). Longer plasmid constructs (1873bp) that we used in dual luciferase reporter assays were predicted to have five GRE’s, while two GREs were predicted for the shorter plasmid constructs (785 bp). One of these GRE lies within the region encompassing variant rs10764881 and indicates that perhaps this variant could disrupt binding. There are no other SNPs that fall near to any GRE in this region except for rs2782888 (non-polymorphic in Caucasians but has a MAF of 8% in an African population, Supplementary Fig. 4). Normally, after glucocorticoid receptor-steroid binding occurs, this complex is transported to the nucleus where it can act as a transcription factor enhancing *MGMT* expression. One could hypothesize that rs10764881 allele A disrupts this binding and results in less efficient *MGMT* transcription. By treating HEK cells containing the transfected plasmids with dexamethasone we were able to confirm that the plasmid containing SNP rs10764881 A allele demonstrated significantly lower expression levels (*p* = 0.0007) compared to the rs10764881 G allele construct, in spite of the number of GREs present. Previous studies confirmed the inducible effects of dexamethasone on *MGMT* mRNA and protein levels through glucocorticoid binding sites [27]. These results were further confirmed by performing an EMSA (Supplementary Fig. 5). However, in EMSA the protein-DNA band did not disappear completely when the competitive control probe was added suggesting that there could be non-specific binding or other transcription factors that may compete for this site.

One of the limitations of our study is the size of the exploratory cohort that was small and therefore lacked the statistical power to truly reject false negatives. Nevertheless, the significance of *MGMT* rs10764881 association in an extended cohort indicates true nature of this association. In in vitro *MGMT* knockout studies neither promoter methylation pattern nor the activity of MGMT in both parent and knockout HAP1cells was measured, Further, multiple clones with and without MGMT expression were not tested that might explain no significant difference in BU IC50 values between cells with and without *MGMT*. Super shift assays with GRE specific antibodies was also not incorporated in EMSA experiments. The incidences of aGvHD between exploratory and extended cohort were also different, however, the observed association remained significant in the extended cohort. The difference in the observed aGvHD incidences between the exploratory and extended cohorts could be attributed to the differences in the distribution of the stem-cell source, conditioning regimen used and aGvHD prophylaxis among the patients between the cohorts. These differences could also be attributed to center specific practices.

It is known that aGvHD grades 2–4 reduces the risk of relapse in pediatric HSCT, especially in acute lymphoblastic leukemias indicating significant graft versus leukemia effect in children in aGvHD. Chronic GvHD was also shown to reduce the risk of relapse mostly in acute myeloid leukemias (AML), with no survival advantage in both scenarios [28]. We did not observe an association of the *MGMT* rs10764881 with cumulative incidence of relapse. This may possibly be explained by higher percentage of AML cases in the cohort, varying disease status (heterogeneity) at the time of HSCT and partly explained by the altered expression profiles of the *MGMT* and its regulation by methylation status of its promoter in cancer cells. In general, cancer tissues exhibit increased MGMT activity compared to that of normal tissues [29], however, concordance between MGMT activity and clinical outcomes in HSCT setting remains to be determined. Several reports in pediatric brain tumors have shown, that there is higher MGMT activity compared to that seen in adults, responded poorly to the alkylating agent temozolamide, and silencing MGMT in such tumors resulted in better response [20] MGMT activity is also determined by hypermethylation of its promoter that results in lower expression of MGMT [30]. We could not investigate the methylation status of the *MGMT* promoter in this study as varying exposures to various chemotherapy drugs prior to the HSCT in children might have had an impact on the methylation status of the *MGMT* promoter, that may be more apparent in malignant cells (modulating the clinical outcomes such as relapse) than in the normal cells. The relevance of this genetic variant association in relation to the promoter methylation needs to be investigated in future.

To conclude, children receiving BU-based myeloablative conditioning prior to allo-HSCT and carrying *MGMT* rs10764881 variant are at increased risk of developing aGvHD 2–4. We hypothesize that children with efficient MGMT function are at lower risk of aaGvHD2–4 possibly by reducing the activation of Phase 1 of the aGvHD cascade. *MGMT* rs10764881 should be validated in an independent cohort, as a predictive marker of aGvHD, in combination with other associated cytokine polymorphisms and non-genetic factors of the host to perform a pre-transplant acute aGvHD risk assessment.

## Methods

### Patient sample

#### Exploratory cohort

Sixty children who underwent an allo-HSCT after myeloablative conditioning with BU/CY from 2001 to 2010 at CHU Saint-Justine, Montreal, Canada. This sample was used to genotype fifty-one chosen SNPs within seventeen DNA-repair genes.

#### Extended cohort

The study was extended to *n* = 187 by including 122 children who had undergone allo-HSCT at five different centers (Supplementary Table 3). They were genotyped for those SNPs that showed a significant association within the exploratory sample after accounting for multiple testing correction (see Table 1 for the patient’s characteristics). The Institutional Review Board at each center approved the study and all patients and/or parents provided informed consent. Details of inclusion criteria are available at Clinicaltrials.gov site (NCT01257854) and the Australian New Zealand Clinical Trials registry (ACTRN12612000544875).

##### Treatment

intravenous. BU (Busulfex^®^, Otsuka Pharmaceuticals, Saint-Laurent, Montreal, QC, Canada or Busilvex^®^, Pierre Fabre Laboratory, Paris, France) administration was given as a 2 h or 3 h infusion depending on whether the patients were given four times daily or once daily dose, respectively. BU first dose was either age or weight-based and pharmacokinetic (PK) guided dose adjustment was performed in order to obtain a cumulative AUC of 57.6–86.4 mg*h/L (Supplementary Table 1). Co-medication to BU, aGvHD prophylaxis and serotherapy are summarized in Table 1. All patients received non-manipulated grafts.

##### Clinical outcomes

aGvHD was graded according to established grading criteria [31] and considered up to day 180 post HSCT [32].

##### Genotyping

Peripheral blood was collected prior to myeloablation and the DNA extracted using a DNA extraction kit (FlexiGene DNA kit, Qiagen GmbH, Hilden). Seventeen genes in total were chosen for investigation (Table 2). We selected 17 candidate genes through a literature search from key protein-coding genes in simple demethylating repair pathways (*MGMT, ALKBH1)*, BER pathway genes such as APEX1, LIG1, LIG4, and XRCC1 or double-strand break repair pathways mainly associated with DNA cross-linking by bifunctional alkylating agents such as BU (*ATM, BRCA1, EXO1, FAN1, FANCD2, MRE11, NBN, RAD50, RAD51, RFC1*). See Supplementary Material Methods for details on variant selection criteria.

##### Statistics analysis

All statistical analyses were performed using SPSS software version 25 (IBM Corp) and R statistical software version 3.6.2. Association analysis was performed with individual polymorphisms that were in HWE and compared to the HapMap Caucasian population MAF data.

##### Univariate analysis

The SNPs were tested for association with aGVHD 2–4 in the exploratory cohort; using a *X*^2^ test in a model (additive or dominant) that best fit the data according to genotype frequency among cases and controls. SNPs with a *p* value below a 0.001 cut off were retained for further analysis according to the Bonferroni correction. Incidence of aGvHD 2–4 was estimated using cumulative incidence function within competing risk package (cmprsk) using R with death occurring before aGvHD as a competing risk and compared using Grays’ test [33] for significant associations. Clinical characteristics tested in the univariate analysis in the extended cohort were HLA compatibility (matched related donor vs. other donors); stem-cell source (bone marrow; peripheral blood stem cells; cord blood); conditioning regimen (based on the number of alkylating agents, one versus 2 or more,); underlying disease (malignant; non-malignant); *GSTA1* metabolic capacity based on diplotypes (classified as slow vs. rapid and normal metabolizers);); serotherapy (not received versus received). aGvHD prophylaxis was not assessed separately in the statistical model as it was highly associated with the stem-cell source. Variables with *p* value <0.1 were retained for multivariable analysis. Clinical characteristics between the exploratory and extended cohorts were compared using *X*^2^ test (categorical) or Mann–Whitney *U* test (continuous variables).

##### Multivariable analysis

Significant SNPs were subsequently re-analysed by competing risk analysis to compare the cumulative incidence of aGvHD 2–4. If still significant, they were retained for estimating the Hazard Ratios with a 95% confidence interval (CI) in Cox-regression multivariable analyses with additional risk factors (using a backward stepwise conditional method). Risk factors in the multivariable analysis included: HLA compatibility, stem-cell source; conditioning regimen, use of serotherapy; baseline disease; *GSTA1* metabolic capacity based on diplotypes [9]. Two additional PK measures were included as continuous variables: first day BU AUC and cumulative BU AUC. Both have previously been reported in relation to toxicity [5, 9]. Additionally, the latter was included in order to control for the variation in the target AUC across the conditioning regimens. Multinomial logistic regression was used to assess the role of the associated SNPs on aGvHD severity, by considering aGvHD grade 1 as reference. For cellular assays, inhibitory concentration 50 (IC50) was determined by nonlinear curve fitting of percent cell survival against concentrations of BU for each cell line in GraphPad Prism, version 7.02.

### Investigations examining the functional role of the associated gene and variant(s)

#### *MGMT* mRNA expression pre and post Busulfan exposure

22 CEPH lymphoblastoid cell lines (Coriell Institute, New Jersey, USA) were obtained with known genotypes extracted from the 1000 genome project (https://www.ncbi.nlm.nih.gov/variation/tools/1000genomes) and used for mRNA expression differences pre and post BU exposure (see Supplementary Material Methods for details).

#### Cell viability studies on HAP1 *MGMT* knockouts

To understand the cellular sensitivity of a cell when *MGMT* is knocked out and after exposure to BU, the near-haploid human cell line HAP1 (Horizon discovery, Cambridge, UK) was used. One Human HAP1 parental control cell line (C631) and one human *MGMT* knock out, edited by CRISPR/Cas9 to contain a 20 bp deletion in a coding exon of *MGMT* (HZGHC000430c006). These cell lines were treated (at passage 2–4) with BU concentrations (25, 50, 100, 250, and 500 µM) for 48 h. Experiments were performed in triplicate on three occasions. Real-Time Cell growth inhibition was evaluated using the CellTiter 2.0 assay (Promega Corporation, 2800 Woods Hollow Road, Madison, USA).

### Dual luciferase reporter-gene assays

Functionality of the rs10764881 or for other SNPs that are in LD with this SNP were assessed by site-specific mutations in *MGMT* promoter pGL4.10 luciferase reporter plasmid constructs (1.8 kb). Luciferase reporter activity was measured by transfecting primary keratinocytes (see Supplementary Material Methods).

#### Electrophoretic mobility shift assay (EMSA)

EMSA was performed to test the *MGMT* promoter DNA-protein binding (transcription factor) capacity of the selected region and if it is influenced by the presence or absence of variant (see Supplementary Material Methods for details).

## Supplementary information

Supplementary Figure 1

Supplementary Figure 2

Supplementary Figure 3

Supplementary Figure 4

Supplementary Figure 5

Supplementary Table 1

Supplementary Table 2

Supplementary Table 3

Supplementary Methods
