# Supplementary material for: Association study of candidate DNA-repair gene variants and acute graft versus host disease in pediatric patients receiving allogeneic hematopoietic stem-cell transplantation
Source: Pharmacogenomics J. 2021 Oct 28;22(1):9–18. doi: 10.1038/s41397-021-00251-7 (PMC8794787; doi:10.1038/s41397-021-00251-7)
Supplement: Supplementary file 1 — Supplementary Figure 1 [file 41397_2021_251_MOESM1_ESM.docx]

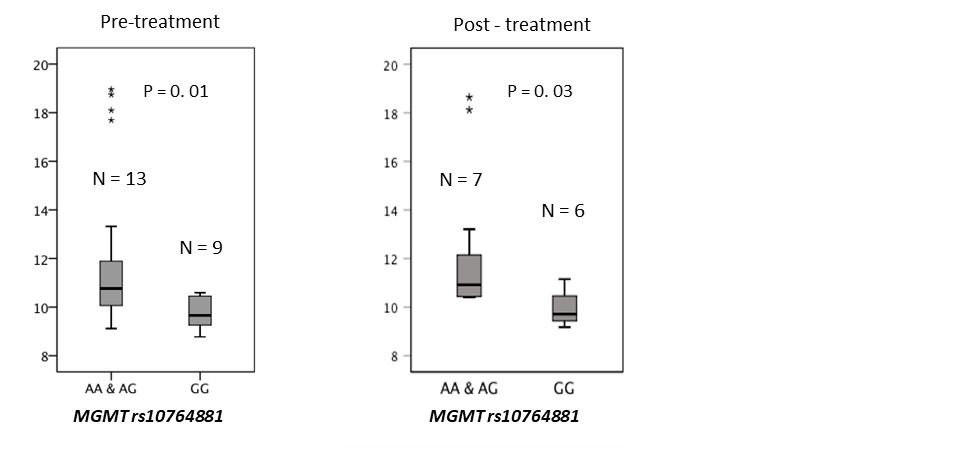


**Supplementary Figure 1. mRNA expression levels of *MGMT* in Lymphoblastoid Cell Line pairs at baseline dependent on variant genotype**. Illustrates the expression differences of the SNPs that showed significant association in the clinical study with aGvHD. For the pre-treatment analysis 22 LCL were investigated, while for the post-treatment only 17 LCLs were investigated, for the repeated measures analysis 13 pairs of LCL were compared. *MGMT* rs10764881 demonstrated significant expression differences indicated as Ct values (y-axis) dependent on the genotype (x-axis) before and after Bu treatment in the same direction. Genotype GG demonstrated higher expression levels at baseline (pre-treatment) and post treatment. Baseline fold changes for GG resulted in being 1.5 times higher than AA/AG carriers. Post treatment differences were slightly lower, 1.2-fold change.
