# Supplementary material for: Association study of candidate DNA-repair gene variants and acute graft versus host disease in pediatric patients receiving allogeneic hematopoietic stem-cell transplantation
Source: Pharmacogenomics J. 2021 Oct 28;22(1):9–18. doi: 10.1038/s41397-021-00251-7 (PMC8794787; doi:10.1038/s41397-021-00251-7)
Supplement: Supplementary file 2 — Supplementary Figure 2 [file 41397_2021_251_MOESM2_ESM.docx]

**
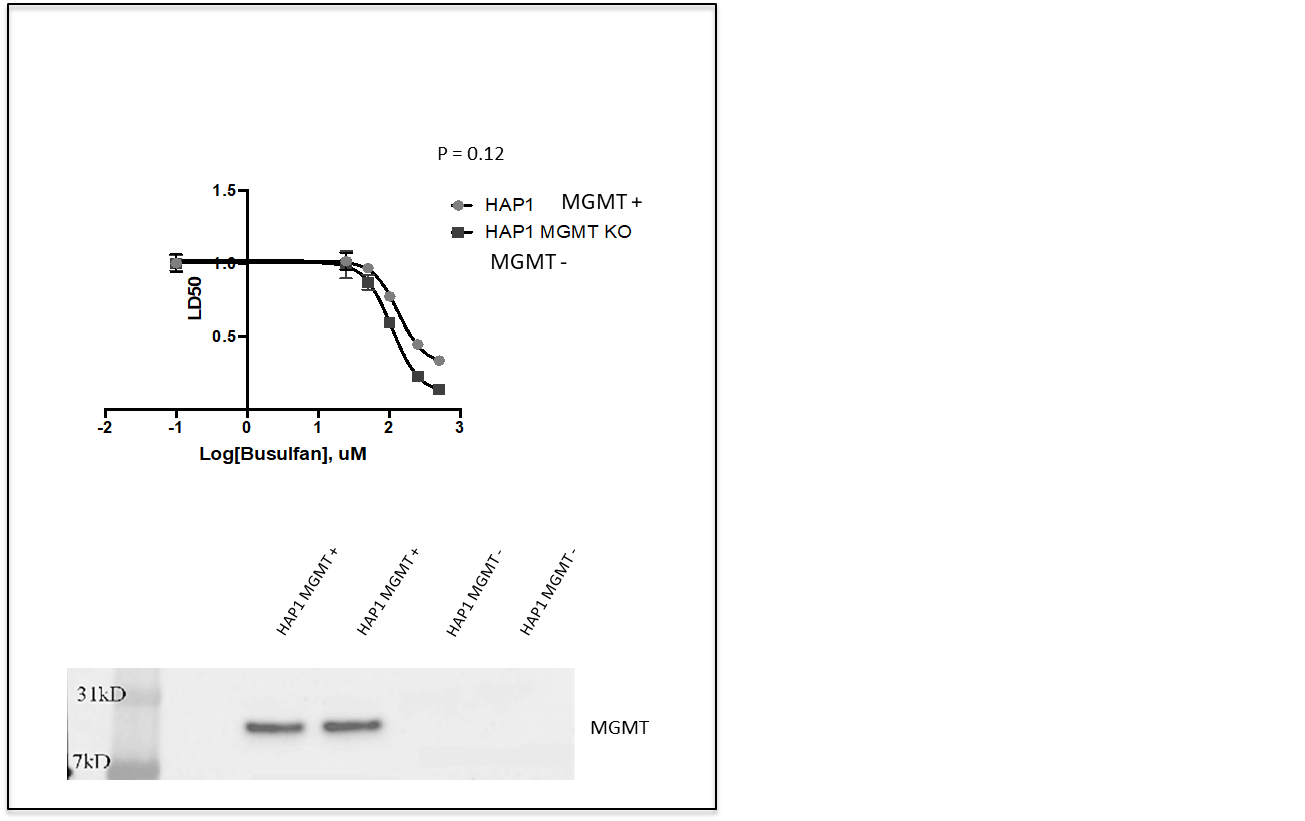
**

**Supplementary Figure 2. HAP1 *MGMT* Knockout study and BU exposure.** Illustrates the sensitivity of HAP1 cells after BU exposure. HAP1 MGMT knockout cells demonstrated a mean IC50 = 102.21uM +/- 6.4uM) compared to the HAP1 parental that demonstrated a mean IC50 = 117.68uM +/- 16.7uM).x-axis represents BU concentration in µM and y-axis represents percentage cell viability. IC50 values were obtained by nonlinear curve fitting. At the base of this figure is a western blot confirming that MGMT was knocked out from the HAP1 non-parental cells.
