# Supplementary material for: Association study of candidate DNA-repair gene variants and acute graft versus host disease in pediatric patients receiving allogeneic hematopoietic stem-cell transplantation
Source: Pharmacogenomics J. 2021 Oct 28;22(1):9–18. doi: 10.1038/s41397-021-00251-7 (PMC8794787; doi:10.1038/s41397-021-00251-7)
Supplement: Supplementary file 3 — Supplementary Figure 3 [file 41397_2021_251_MOESM3_ESM.docx]

N=38

N=20

p=0.02

**Ln BU IC50 (µM)**


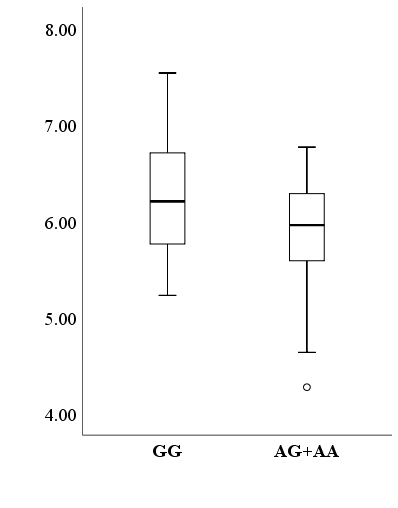


***MGMT* rs10764881 genotypes s10764881**

**Supplementary Figure 3.** Busulfan IC50 values in lymphoblastoid cells carrying different genotypes (dominant model) at *MGMT* rs10764881. The number of cells in each group and the p value (two-tailed) are presented on the plot.
