# Supplementary material for: Association study of candidate DNA-repair gene variants and acute graft versus host disease in pediatric patients receiving allogeneic hematopoietic stem-cell transplantation
Source: Pharmacogenomics J. 2021 Oct 28;22(1):9–18. doi: 10.1038/s41397-021-00251-7 (PMC8794787; doi:10.1038/s41397-021-00251-7)
Supplement: Supplementary file 4 — Supplementary Figure 4 [file 41397_2021_251_MOESM4_ESM.docx]

1 CATGTTTCAT CTGTCCTTCC ACATTAGTTT AAATGTGAAT GGCTGCTTGGAACACAGTGT

61 TGA**G**AAGGAT TCCGAGGCTG TGTCCAGGTT CAGTGGGGAA AAAGTGCCTT GATTTGACCA

121 TTATATCGTG TACTCAGGGC CTAGTTTATT TCCCACAAGC AGCTATTTAA AACATGTCTC

181 ATCCAAGGCA GGGTCGGGGT GGGGAACCTC AGAGGATCAA TGATGCAGCC TGTATTGTCA

241 CCAGGGCTCA TCCCAAAGCA AATCCTGCTG AAGTTCTCCT CTGAACAACC CTTATCACTC

301 ATGCTAATAT GTTGGATCCT GCAAGTCACA AATACGAAGG TATGAGCATT GGCCGAATCC

361 CAACAGGGAA ACTTCACCGG GAAGGTGAAG ACCCCTTTCC ATTAAGATAA CAAACAGAAC

421 CTAGAAGGCC CGTAGCATGG TGGCTTAGAA CATGTGGGCC TTGGATGGGA TCCTGCCAAG

481 GGGTGTGTGA CCTCTCCGAA GCCTCCAGGG ATGATACTAC TCCCTGGGGT GCTTATGCCA

541 ACCACGTTAG AGACAATGGT TTCTGTACCC ATTGCCTGGG GCTGCCATAA TAAAGTGCCA

601 CACACTGAGT GGCTTAAAAC AACAGAAACC TATTGTCTCA CACTT**C**CGGG GGCCAGAAGT

661 TTGAAACCCA GGTGTGTTAG GATCCTGCTC CCTCTGAAGG CTCCAGGGAA GAGTGTCCTC

721 TGCTCCCTCC GAAGGCTCCA GGGAAGGGTC TGTCCTCTTA GGCTTCTGGT GGCTTGCAGG

781 TGCAGCCCTC CAATCCTCCT CCCCAAGCGG CCT**T**CTGCCT ATAAGGACAC GAGTCATACT

841 GGATGAGGGG CCCACTAATT GATGGCTTCT GTAAAGTCCC CATCTCCAAA TAAGGTCACA

901 TTGTGAGGTA CTGGGAGTTA GGACTCCAAC ATAGCTTCTC TGGTGGACAC AATTCAACTC

961 CTAATAACGT CCACACAACC CCAAGCA**G**GG CCTGGCACCC TGTGTGCTCT CTGGAGAGCG

1021 GCTGAGTCAG GCTCTGGCAG TGTCTAGGCC ATCGGTGACT GCAGCCCCTG GACGGCATCG

1081 CCCACCACAG GCCCTGGAGG CTGCCCCCAC GGCCCCCTGA CAGGGTCTCT GCTGGTCTGG

1141 GGGTCCCTGA CTAGGGGAGC GGC**A**CCAGGA GGGGAGAGAC TCGCGCTCCG GGCTCAGCGT

1201 AGCCGCCCCG AGCAGGACCG GGATTCTCAC TAAGCGGGCG CCGTCCTACG ACCCCCGCGC

1261 GCTTTCAGGA CCACTCGGGC ACGTGGCAGG TCGCTTGCAC GCCCGCGGAC TATCCCTGTG

1321 ACAGGAAAAG GTAC**G**GGCCA TTTG**G**CAAAC TAAGGCACAG AGCCTCAGGC GGAAGCTGGG

1381 AAGGCGCCGC CCGGCTTGTA CCGGCCGAAG GGCCATCCGG GTCAGGCGCA CAGGGCAGCG

1441 GCGCTGCCGG AGGAC**C**AGGG CCGGCGTGCC GGCGTCCAGC GAGGATGCGC AGACTGCCTC

1501 AGGCCCGGCG CCGCCGCAC**A** GGGCATGCGC CGACCCGGTC GGGCGGGAAC ACCCCGCCCC

1561 **T**CCCGGGCTC CGCCCCAGCT CCGCCCCCGC GCGCCCCGGC CCCGCCCCCG CGCGCTCTCT

1621 **T**GCTTTTCTC AGGTCCTCGG CTCCGCCCCG CTCTAGACCC CGCCCCACGC CGCCATCCCC

1. rs10764881, alleles G/A, upstream gene variant, position 10:129465567
2. rs36003727, alleles C/T, upstream gene variant, position 10: 129466150
3. rs1711646, alleles T/G, upstream gene variants, position 10: 129466317
4. rs74162154, alleles G/C, upstream gene variants, position 10: 129466491
5. rs1625649, alleles A/C, upstream gene variants, position 10: 129466667
6. rs113813075, alleles C/A, upstream gene variant, position 10: 129466757
7. rs79442343, alleles G/A, upstream gene variant, position 10: 129466838
8. rs34180180, alleles G/A, upstream gene variant, position 10: 129466848
9. rs34138162, alleles C/A, upstream gene variant, position 10: 129466959
10. rs1623007, alleles A/T, upstream gene variant, position 10: 129467023
11. rs2782888, alleles T/G, upstream gene variant, position 10: 129467064,
12. rs181536588, alleles T/A, upstream gene variant, position 10: 129467124

**Supplementary Figure 4. Glucocorticoid binding element sites in the promoter and upstream to *MGMT***. Glucocorticoid binding elements (highlighted in grey) in the promoter region of *MGMT*. Calculated using the MatInspector online program. Relevant SNPs are illustrated too. At position 64 lies rs10764881 identified as number 1.
