# Supplementary material for: Association study of candidate DNA-repair gene variants and acute graft versus host disease in pediatric patients receiving allogeneic hematopoietic stem-cell transplantation
Source: Pharmacogenomics J. 2021 Oct 28;22(1):9–18. doi: 10.1038/s41397-021-00251-7 (PMC8794787; doi:10.1038/s41397-021-00251-7)
Supplement: Supplementary file 5 — Supplementary Figure 5 [file 41397_2021_251_MOESM5_ESM.docx]

**
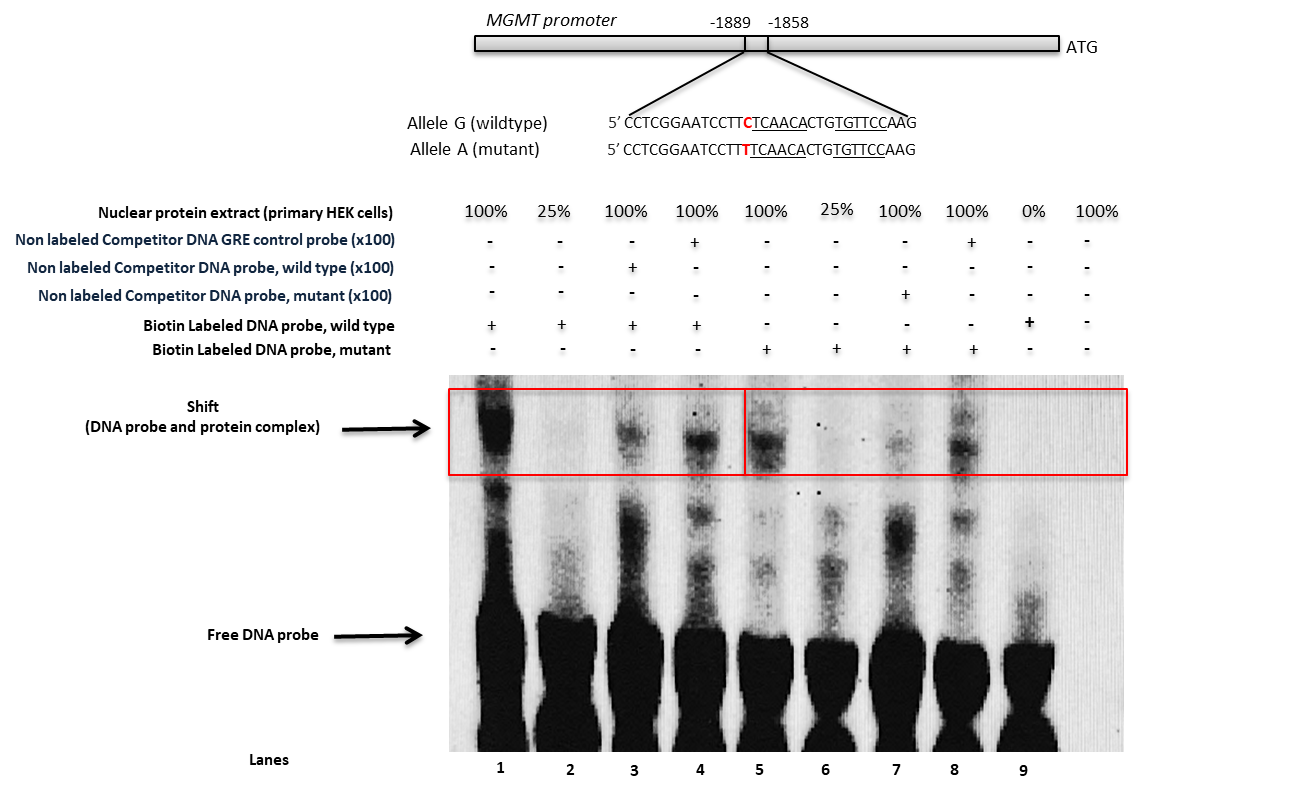
**

**Supplementary Figure 5. Electrophoretic Mobility Shift Assay examining DNA – protein binding sites near *MGMT* rs10764118.** Electrophoretic Mobility Shift Assay (EMSA) Illustrating at the top of the gel picture the core consensus sequence underlined (5’TCAACA-3’) identified by the MatInspector as a predicted GRE half-site, and a half GRE site (5’TGTTCC-3’) reported by Del Monaco in 1997 (29). In red is the base change and position of *MGMT* SNP rs10764881. The polyacrylamide gel shows from left to right, that a shift is established when nuclear human keratinocyte (HEK) protein is incubated with varying concentrations of both labelled MatInspector predicted probes (lane 1 and 2, for wildtype allele G and lane 5 and 6, for mutant allele A) with higher binding to the wild type probe. Furthermore, the intensity of the shift diminished when both the competitive MatInspector predicted probe (lane 3 and 7) and the GRE competitive control probe (lane 4 and 8) was added at x100 concentration (non-labelled). Lane 9 contains only labeled probe so no shift is visible, while in lane 10 no protein was added thus no bands are visible as expected.
