# Supplementary material for: Association study of candidate DNA-repair gene variants and acute graft versus host disease in pediatric patients receiving allogeneic hematopoietic stem-cell transplantation
Source: Pharmacogenomics J. 2021 Oct 28;22(1):9–18. doi: 10.1038/s41397-021-00251-7 (PMC8794787; doi:10.1038/s41397-021-00251-7)
Supplement: Supplementary file 6 — Supplementary Table 1 [file 41397_2021_251_MOESM6_ESM.docx]

**Supplementary Table 1. Distribution of *MGMT* rs10764881 in varying aGvHD severity**

|  | **AA & AG (Variant)** | **GG**  **(Normal)** | **Total** |
| --- | --- | --- | --- |
| **No aGvHD** | 32 (35%) | 59 (65%) | 91 |
| **aGvHD 1** | 30 (57%) | 22 (42%) | 52 |
| **aGvHD 2-4** | 26 (66%) | 13 (33%) | 39 |
| **Total** | 88 | 94 | 182 |

Frequency and percentage (in brackets) of aGvHD incidences are shown for the entire cohort dependent on rs10764881 genotype (AA & AG vs GG). Multinomial regression analyses demonstrated no significant difference between aGvHD 1 vs aGvHD 2-4 data (p=0.3).
