# Supplementary material for: Association study of candidate DNA-repair gene variants and acute graft versus host disease in pediatric patients receiving allogeneic hematopoietic stem-cell transplantation
Source: Pharmacogenomics J. 2021 Oct 28;22(1):9–18. doi: 10.1038/s41397-021-00251-7 (PMC8794787; doi:10.1038/s41397-021-00251-7)
Supplement: Supplementary file 7 — Supplementary Table 2 [file 41397_2021_251_MOESM7_ESM.docx]

**Supplementary Table 2. Allele frequencies of *MGMT* rs10764881 in major populations**

| **GnomAD data [1]** | **rs10764881** | **rs10764882** | **rs1625649** | **rs1625649** |
| --- | --- | --- | --- | --- |
| **Population** | **Allele "G"frequency (count)** | **Allele "A" frequency (count)** | **Allele "C"frequency (count)** | **Allele "A"frequency (count)** |
| African/African-American | 0.905 (37994) | 0.095 (4004) | 0.494 (20685) | 0.506 (21213) |
| Amish | 0.655 (588) | 0.345 (310) | 0.651 (586) | 0.349 (314) |
| Latino/Admixed American | 0.691 (9432) | 0.309 (4208) | 0.639 (8729) | 0.361 (4925) |
| Ashkenazi Jewish | 0.690 (2290) | 0.310 (1028) | 0.318 (1056) | 0.682 (2262) |
| East Asian | 0.672 (2105) | 0.328 (1027) | 0.690 (2156) | 0.310 (968) |
| European (Finnish) | 0.711 (7428) | 0.289 (3012) | 0.631 (6554) | 0.369 (3826) |
| European (NON-Finnish) | 0.683 (44085) | 0.317 (20449) | 0.660 (42547) | 0.340 (21931) |
| Other | 0.747 (1603) | 0.253 (543) | 0.623 (1339) | 0.377 (811) |
| Souith Asian | 0.712 (2159) | 0.288 (875) | 0.661 (2011) | 0.339 (1033) |
| **All populations Combined** | **0.752 (107684)** | **0.248 (35456)** | **0.608 (86869)** | **0.392 (56077)** |
|  |  |  |  |  |
| **1000 genomes data [2]** | **rs10764881** | **rs10764881** | **rs1625649** | **rs1625649** |
| **Population** | **Allele "G"frequency (count)** | **Allele "A" frequency (count)** | **Allele "C"frequency (count)** | **Allele "A"frequency (count)** |
| African | 0.954 (1261) | 0.046 (61) | 0.477 (630) | 0.523 (692) |
| American | 0.669 (464) | 0.331 (230) | 0.610 (423) | 0.390 (271) |
| East-Asian | 0.683 (688) | 0.317 (320) | 0.669 (674) | 0.331 (334) |
| European | 0.691 (695) | 0.309 (311) | 0.657 (661) | 0.343 (345) |
| South Asian | 0.677 (662) | 0.323 (316) | 0.615 (601) | 0.385 (377) |
| **All populations combined** | **0.753 (3770)** | **0.247 (1238)** | **0.597 (2989)** | **0.403 (2019)** |
