# Supplementary material for: Association study of candidate DNA-repair gene variants and acute graft versus host disease in pediatric patients receiving allogeneic hematopoietic stem-cell transplantation
Source: Pharmacogenomics J. 2021 Oct 28;22(1):9–18. doi: 10.1038/s41397-021-00251-7 (PMC8794787; doi:10.1038/s41397-021-00251-7)
Supplement: Supplementary file 8 — Supplementary Table 3 [file 41397_2021_251_MOESM8_ESM.docx]

**Supplementary Table 3. Busulfan Cross calibration study and Pharmacokinetic estimation and parameters per Centre**

| **Centers (number of patients recruited in extended cohort)** | **Busulfan Initial IV Dose** | **Busulfan, Dose adjusted** | **Method of Busulfan concentration** | **The theoretic adjusted Busulfan doses** | **The determination of pharmacokinetic parameters, estimated from the first dose.** |
| --- | --- | --- | --- | --- | --- |
| **CHU St-Justine, Montreal, Quebec Canada (n=39)** | 0.8mg/kg/dose (infants≥3months and <1 year of age; 1 mg/kg/dose (children≥1 year and <4 years old); 0.8mg/kg/dose (children≥4 years old) | Dose adjustment occurred from the fifth dose onwards if 4 x daily or after the 1^st^ dose for 1 x daily administration. | HPLC/LC-MS/MS | Based on a target AUC of 57.6 -86.4 mg*h/L | Non-compartmental analysis (WinNonlin, version 3.1, Pharsight) |
| **Geneva University Hospital, Switzerland (n= 10)** | 0.8mg/kg/dose (infants≥3months and <1 year of age; 1 mg/kg/dose (children≥1 year and <4 years old); 0.8mg/kg/dose (children≥4 years old) | Dose adjustment occurred from the fourth dose onwards | LC-MS/MS | Based on a target Css of 57.6 -86.4 mg*h/L | As in row 1 |
| **Leiden**  **University Medical Center Netherlands (n=12 )** | 0.8 to 1mg/kg/dose (infants≥4 years old);1mg/kg/dose (infants<4 years old) | Dose adjustment occurred at the fifth or ninth dose | HPLC/LC-MS/MS | Dose adjustment was allowed to a limit of 1.0mg/kg every 6 hours if the target AUC of 4.93 mg hr/L was not achieved. Then was adjusted only when the AUC differed by > 10% from the target AUC | As in row 1 |
| **Robert Debré University Hospital, Paris France (n=9)** | <9kg (1mg/kg/dose); 9≥to<16kg (1.2mg/kg/dose); 16≥to≤23kg (1.1mg/kg/dose); >23to≤34kg (0.95mg/kg/dose); >34kg (0.8mg/kg/dose) | Dose adjustment occurred at the seventh or ninth dose | GC-MS | Based on a target Css of 57.6 - 98.6 mg*h/L | As in row 1 |
| **The Children’s Hospital at Westmead, NSW, Australia (n=52)** | < 6 months (3.2 mg/kg/dose)  <9kg (4mg/kg/dose); 9≥to<16kg (4.8mg/kg/dose); 16≥to≤23kg (4.4mg/kg/dose); >23to≤34kg (3.8mg/kg/dose); >34kg (3.2mg/kg/dose) | Dose adjustment occurred from the fifth dose onwards if 4 x daily or after 1^st^ dose for 1 x daily dose administration | GC-ECD | Myeloablative target for a cumulative AUC of 75 ± 5 mg/L.h | Non-compartmental analysis (Kinetica Version 4.0) |

**Pharmacokinetic Parameters:** A cross calibration study was conducted by our group with the collaboration of Pierre Fabre Laboratories between all centers participating in the study to validate analytical method (data available upon request). Only centers with the measured BU concentrations falling within ± 20% of the theoretical concentrations were included for analysis of PK parameters. Abbreviations: AUC: Area under the curve; Cl: Clearance; Css: Steady- state concentration; GC-MS: Gas Chromatography Mass Spectrometry; GC-ECD: Gas Chromatography with Electron Capture Detector; HPLC: High Performance Liquid Chromatography; LC-MS/MS: Liquid chromatography–mass spectrometry
