# Supplementary material for: Association study of candidate DNA-repair gene variants and acute graft versus host disease in pediatric patients receiving allogeneic hematopoietic stem-cell transplantation
Source: Pharmacogenomics J. 2021 Oct 28;22(1):9–18. doi: 10.1038/s41397-021-00251-7 (PMC8794787; doi:10.1038/s41397-021-00251-7)
Supplement: Supplementary file 9 — Supplementary Methods [file 41397_2021_251_MOESM9_ESM.docx]

**Supplementary Methods:**

**Candidate genes:** Candidate genes coding for key proteins of demethylating repair pathways (*MGMT, ALKBH1)*, BER pathway genes such as *APEX1, LIG1, LIG4* and *XRCC1* or double strand break repair pathways mainly associated with DNA cross linking bifunctional alkylating agents such as BU (*ATM, BRCA1, EXO1, FAN1, FANCD2, MRE11, NBN, RAD50, RAD51, RFC1*) were selected for the investigation [3,4,5,6].

**Genotyping:**

Single nucleotide polymorphisms (SNPs) in these genes were identified from the HapMap project using the genome variation server 5.11 (available at: <http://gvs.gs.washington.edu/GVS/>). A 1,000bp boundary was set either side of each gene before searching for SNPs with a minimal allele frequency above 5% (Caucasian population). The SNPs were tagged with r2 threshold of 0.80, 80% data coverage for tagSNPs and 70% data coverage for clustering. Priority was given to potential functional SNPs (Table 2 in main text) and SNPs within exonic, promoter and 3’UTR regions, however if none where identified with an acceptable minor allele frequency (MAF) or the region appeared difficult to PCR, then intronic SNPs, which provided the tagging of the priority regions were selected. Few SNPS were included even though their MAF is below 5% because they are non-synonymous SNPs or predicted to be damaging by the function prediction tools (rs17825440, rs28897687) or are potentially regulatory SNPs (rs3136814). The final SNPs (N = 51) chosen for genotyping in exploratory cohort are summarized in Table 2 of the main text. Genotyping was centrally performed at the Research platform for paediatric oncology and haematology in Geneva, with the StepOne Plus real time PCR (Applied Biosystems, Foster City, CA, USA) using TaqMan allele discrimination assays with nucleotide specific probes or using pre-designed TaqMan SNP genotyping assays.

***MGMT* mRNA expression pre and post Busulfan exposure:**

The lymphoblastoid cell lines (n=22) were maintained in Roswell Park Memorial Institute (RPMI) 1640 medium with 10% heat inactivated foetal bovine serum and 1% Penicillin–Streptomycin (Sigma; P0781). BU (Sigma), was reconstituted as follows: 61.6 mg of BU was dissolved in 1 mL dimethylsulfoxide (DMSO; Sigma) at 250 mM and stored at -20^o^C. This agent was added directly from the stocks to the exponentially growing Lymphoblast cells to obtain a final BU concentration of 500µM. This concentration was calculated to be the average IC50 for LCLs (data not shown). The maximal final concentration of DMSO in the medium did not exceed 0.1%. After 48 hrs, cells were washed with PBS, centrifuged and pellets collected for later extraction of RNA. Total RNA from the cells was extracted using the Purelink RNA kit (Life technologies) and converted to cDNA using Superscript VILO (Life technologies) and stored at -80^0^C until further analysis. Quantification of the mRNA was performed before the downstream applications using nanodrop UVS-99 microvolume bio spectrophotometer (UVS). Gene expression data of the selected DNA repair gene, prior to BU exposure and after BU treatment was generated using RT-PCR (Step One plus Real time PCR system from Applied Biosystems, Foster City, USA) and SYBR green chemistry (SYBR**®** Select Master Mix, Applied Biosystems, Switzerland) with *GAPDH* gene as an internal control. The primers used for *MGMT* were: forward: 5'-ACCGTTTGCGACTTGGTACTT-3'; reverse: 5'-GGAGCTTTATTTCGTGCAGACC-3'. The gene Ct values (cycle threshold) for *MGMT* were corrected by the efficiency (R^2 =^ 0.99, 100%) and normalized to the internal control. The relative gene expression was calculated ΔΔCt method. Comparisons were performed overall for *MGMT* pre and post BU exposure and between gene variant (rs10764881 AA/AG vs. GG) groups at pre and post BU treatment.

***In vitro* cellular sensitivity to Busulfan**

We obtained 58 HapMap CEU samples from Coriell Institute for Medical Research (Camden, NJ, USA). Cell lines were maintained in RPMI 1640 media supplemented with 15% foetal bovine serum and 1% l-glutamine. The cell lines were passaged two-three times per week at a concentration of 350,000 cells/ml at 37°C in a 95% humidified 5% CO2 atmosphere. Cellular sensitivity to busulfan was measured in these cell lines with increasing concentrations of Busulfan for 48 hours. Cell growth inhibition was evaluated using the CellTiter 2.0 assay (Promega Corporation, 2800 Woods Hollow Road, Madison, WI 53711 USA.

**Association analysis of SNPs with cellular sensitivity (IC50 values) to Busulfan**

IC50 (the concentration required to inhibit 50% of cell growth) was determined by curve fitting of percent cell survival against concentrations of the Busulfan for each cell line in GraphPad Prism, version 7.02 for Windows, GraphPad Software, La Jolla California USA, www.graphpad.com. The IC50 value by curve fitting was acceptable with R2>95% and < 30% of CV between experiments. At least three experimental replicates with acceptable criteria for each cell line were performed [7,8]. Cellular sensitivity to Busulfan (mean IC50 values) among the carriers of MGMT rs10764881 genotypes (dominant model) was compared by using parametric independent t test using log2-transformed IC50 (as the dependent variable). p value (two-tailed) less than 0.05 was considered significant.

**Dual luciferase reporter gene assays to evaluate promoter activity**

To further understand the function of rs10764881 or of other SNPs that are in LD with rs10764881, site-specific mutations for MGMT were designed dependent on the SNPs of interest in the promoter region. Plasmid constructs were prepared by a gene assembly service (GeneScript, Piscataway, USA). Four fragments were cloned into pGl4.10 in front of the firefly luciferase gene (Promega, Maddison, USA) using SacI-XhoI restriction sites. Human epidermal primary keratinocytes (HEK) obtained from ATCC® (ATCC-PCS-200-011) were co-transfected with each of the pGL4.10 MGMT constructs and the pRL-SV40 vector (Promega) that codes for Renilla luciferase for transfection control and normalisation. Promoterless pGL4.10 plasmid (Promega Maddison, USA) was used to determine baseline expression. Transfections of the cells were accomplished by X-tremeGENE™ HP DNA transfection reagent kit (Roche, Basel, Switzerland). Measurement of Luciferase and Renilla activity was determined by Dual luciferase assays (Promega, Maddison, USA) according to the manufacturer’s protocol. Difference in promoter activity between the plasmid constructs was assessed by t-test. Because rs10764881 was predicted by MatInspector [9] to contain a transcription binding site, i.e. a Glucocorticoid responsive element (GRE) and was predicted to lie near to MGMT rs10764881 locus. To investigate if steroid treatment would impact the promoter activity in relation to the variant, HEK cells transfected with the gene reporter plasmids and were stimulated with 0.1µM dexamethasone (Sigma, D8893), for 15hrs and Luciferase reporter activity was examined. The concentrations and timing of dexamethasone exposure were considered and adapted from previous protocols [10,11] to suit HEK cells. Dexamethasone (a glucocorticoid) was dissolved in ethanol at a stock concentration of 10 mM and stored at -20^o^C. This agent was added directly from the stocks to exponentially growing HEK cells.

Electrophoretic mobility shift assay (EMSA)

Thus, nuclear extracts were prepared from Glucocorticoid Receptor-stimulated HEK cells treated with 0.1uM dexamethasone for 15hrs before extraction, using a NE-PER Nuclear and Cytoplasmic Extraction kit (thermo Fisher Scientific Cat No. 78833) as per the manufacturer's instructions. Cell extracts were stored at −80°C until use. Two probes were designed and synthesized by Microsynth containing 5’ fluorescently labeled Dyomics 800 Infrared-Dyes. One MGMT probe contained SNP rs10764881 G (5’ CTTGGAACACAGTGTTGAGAAGGATTCCGAGG); the second contained the minor allele A (5’ CTTGGAACACAGTGTTGAAAAGGATTCCGAGG). Core consensus elements are underlined. An established GRE probe [12] (Santa Cruz 2545), was used as an unlabeled competitor probe (5’ AGAGGATCTGTACAGGATGTTCTAGAT) as well as an unlabeled MGMT probe. Electrophoretic mobility shift assay (EMSA) was performed using a commercially available kit (Thermo Fisher Scientific Cat No. 201848) according to the manufacturer's instructions. Varying concentrations of protein nuclear extracts, with or without 100-fold or 100 excess of unlabeled competitor probe, were incubated for 5 minutes at room temperature in 1 times binding buffer. The Dynomics 781-labeled (20 fmols), double-stranded probes were then added, and the binding reactions were performed for 20 minutes at room temperature. The DNA-protein complexes were resolved on native 6% polyacrylamide gels (Invitrogen Cat No. EC63652), and the protein-DNA complexes were detected using Odyssey Infrared Imaging System (LiCor Biosciences).

**References:**

**1**. Karczewski KJ, Francioli LC, Tiao G, Cummings BB, Alföldi J, Wang Q, Collins RL, Laricchia KM, Ganna A, Birnbaum DP, Gauthier LD, Brand H, Solomonson M, Watts NA, Rhodes D, Singer-Berk M, England EM, Seaby EG, Kosmicki JA, Walters RK, Tashman K, Farjoun Y, Banks E, Poterba T, Wang A, Seed C, Whiffin N, Chong JX, Samocha KE, Pierce-Hoffman E, Zappala Z, O'Donnell-Luria AH, Minikel EV, Weisburd B, Lek M, Ware JS, Vittal C, Armean IM, Bergelson L, Cibulskis K, Connolly KM, Covarrubias M, Donnelly S, Ferriera S, Gabriel S, Gentry J, Gupta N, Jeandet T, Kaplan D, Llanwarne C, Munshi R, Novod S, Petrillo N, Roazen D, Ruano-Rubio V, Saltzman A, Schleicher M, Soto J, Tibbetts K, Tolonen C, Wade G, Talkowski ME; Genome Aggregation Database Consortium, Neale BM, Daly MJ, MacArthur DG. The mutational constraint spectrum quantified from variation in 141,456 humans. Nature. 2020 May;581(7809):434-443. doi: 10.1038/s41586-020-2308-7.

**2**.1000 Genomes Project Consortium, Auton A, Brooks LD, Durbin RM, Garrison EP, Kang HM, Korbel JO, Marchini JL, McCarthy S, McVean GA, Abecasis GR. A global reference for human genetic variation. Nature. 2015 Oct 1;526(7571):68-74. doi: 10.1038/nature15393.

**3**. Casorelli I, Bossa C, Bignami M. DNA damage and repair in human cancer: molecular mechanisms and contribution to therapy-related leukemias. Int J Environ Res Public Health 2012; 9(8): 2636-2657.

**4.** Pallis AG, Karamouzis MV. DNA repair pathways and their implication in cancer treatment. Cancer Metastasis Rev 2010; 29(4): 677-685.

**5**. Kondo N, Takahashi A, Ono K, Ohnishi T. DNA damage induced by alkylating agents and repair pathways. J Nucleic Acids 2010; 2010: 543531.

**6.** Arora M, Lindgren B, Basu S, Nagaraj S, Gross M, Weisdorf D, et al. Polymorphisms in the base excision repair pathway and graft-versus-host disease. Leukemia 2010; 24(8): 1470-1475.

**7.** Jurkovic Mlakar S, Uppugunduri RC, Mlakar V, Huezo-Diaz Curtis P, Nava T, Lesne L, Marino D, Boudal-Khoshbeen M, Aziz Rezgui M, Bittencourt H, Krajinovic M, Ansari M.GSTM1 deletion variant is involved in busulfan response using human lymphoblastoid cell lines. Supplement: Abstracts from the 50th Congress of The International Society of Paediatric Oncology (SIOP). Pediatr Blood Cancer. 2018;65:e27455, https://doi.org/10.1002/pbc.27455, PO-067

**8**. Jurkovic Mlakar S, Uppugunduri RC, Mlakar V, Aziz Rezgui M, Bernard F, Lesne L, Marino D, Duval M, Lo Piccolo R, Chalandon Y, Dupuis L, Schechter T, Bartelink I, Boelens JJ, Bredius R, Dalle JH, Peters C, Bader P, Bittencourt H, Krajinovic M, Ansari M.GST-M1 and -T1 null polymorphisms are associated with lower event-free survival and higher rate of relapse in children with malignancies undergoing allogeneic The 44th Annual Meeting of the European Society for Blood and Marrow Transplantation: Physicians Poster Sessions. Bone Marrow Transplant 53, 145–805 (2019). https://doi.org/10.1038/s41409-018-0354-7; P737

**9.** Cartharius K, Frech K, Grote K, Klocke B, Haltmeier M, Klingenhoff A, et al. MatInspector and beyond: promoter analysis based on transcription factor binding sites. Bioinformatics 2005; 21: 2933-2942.

**10**.Grombacher T, Mitra S, Kaina B. Induction of the alkyltransferase (MGMT) gene by DNA damaging agents and the glucocorticoid dexamethasone and comparison with the response of base excision repair genes. Carcinogenesis 1996; 17: 2329-2336.

**11**.Bindreither D, Ecker S, Gschirr B, Kofler A, Kofler R, Rainer J. The synthetic glucocorticoids prednisolone and dexamethasone regulate the same genes in acute lymphoblastic leukemia cells. BMC Genomics 2014; 15: 662.

**12**.Geng CD, Schwartz JR, Vedeckis WV. A conserved molecular mechanism is responsible for the auto-up-regulation of glucocorticoid receptor gene promoters. Molecular endocrinology 2008; 22: 2624-2642.
